# Optimization of virtual and real registration technology based on augmented reality in a surgical navigation system

**DOI:** 10.1186/s12938-019-0745-z

**Published:** 2020-01-08

**Authors:** Long Chen, Fengfeng Zhang, Wei Zhan, Minfeng Gan, Lining Sun

**Affiliations:** 1 School of Mechanical and Electrical Engineering, Soochow University, Suzhou, 215006 China; 2 Collaborative Innovation Center of Suzhou Nano Science and Technology, Soochow University, Suzhou, 215123 China; 3 Department of Radiation Oncology, The First Affiliated Hospital of Soochow University, Suzhou, China

**Keywords:** Augmented reality, Virtual and real registration, Surgical navigation, Robot, Improved identification method

## Abstract

**Background:**

The traditional navigation interface was intended only for two-dimensional observation by doctors; thus, this interface does not display the total spatial information for the lesion area. Surgical navigation systems have become essential tools that enable for doctors to accurately and safely perform complex operations. The image navigation interface is separated from the operating area, and the doctor needs to switch the field of vision between the screen and the patient’s lesion area. In this paper, augmented reality (AR) technology was applied to spinal surgery to provide more intuitive information to surgeons. The accuracy of virtual and real registration was improved via research on AR technology. During the operation, the doctor could observe the AR image and the true shape of the internal spine through the skin.

**Methods:**

To improve the accuracy of virtual and real registration, a virtual and real registration technique based on an improved identification method and robot-assisted method was proposed. The experimental method was optimized by using the improved identification method. X-ray images were used to verify the effectiveness of the puncture performed by the robot.

**Results:**

The final experimental results show that the average accuracy of the virtual and real registration based on the general identification method was 9.73 ± 0.46 mm (range 8.90–10.23 mm). The average accuracy of the virtual and real registration based on the improved identification method was 3.54 ± 0.13 mm (range 3.36–3.73 mm). Compared with the virtual and real registration based on the general identification method, the accuracy was improved by approximately 65%. The highest accuracy of the virtual and real registration based on the robot-assisted method was 2.39 mm. The accuracy was improved by approximately 28.5% based on the improved identification method.

**Conclusion:**

The experimental results show that the two optimized methods are highly very effective. The proposed AR navigation system has high accuracy and stability. This system may have value in future spinal surgeries.

## Background

With the rapid development of digital image processing, computer vision, network communication and location tracking in recent years, computer-aided surgery (CAS) has emerged as an important component of modern surgical technology [1–4]. The traditional surgical navigation interface was intended only for two-dimensional (2D) observation by doctors; thus, it lacks a display of the total spatial information of the lesion area [5]. Due to the high risk and non-repeatability in the medical field, more surgical guidance and technical assistance are urgently needed. Therefore, CAS has considerable practical significance for studying the application of augmented reality (AR) technology in medical-assisted surgery [6]. Currently, image-guided surgery (IGS) systems have played a very important role in the medical industry [7, 8]. IGS had gradually entered the research field of scientists and has been applied in surgery [9]. This progress indirectly promotes the development of AR in the application field, it can help doctors solve hand–eye coordination problems and achieve accurate stereo spatial positioning and image guidance.

Robots have been extensively employed in many surgical fields [10–12]. Robots have been used to assist in all aspects of spinal surgery, including improving the accuracy of spinal internal fixation, reducing exposure to radiation and improving operating room workflow [13–15]. The use of robots in assisted surgery enables surgeons to make significant improvements in coordination, three-dimensional visualization and fatigue reduction. Minimally invasive spinal surgery (MIS) flourished in the past decade. Robot-assisted spinal surgery was considered an important driving force for the development of minimally invasive surgery in the future. This type of surgery provided patients with smaller incisions and a lower risk of infection during surgery [16–19]. Currently, commercial robotic systems can be divided into passive or active devices [20]. Computer-assisted orthopaedic surgery is a related field of orthopaedic technology development. However, robot-assisted orthopaedic surgery can achieve the accuracy, precision and safety that computer-assisted orthopaedic surgery lacks [21–23].

Augmented reality is a new research field developed by virtual reality (VR). AR is a technology that synthesizes virtual objects generated by computers in real environments [24]. In general, AR described a mediated reality that is typically enhanced by computing devices to enhance the visual perception of the physical real world. Compared with VR, AR was not intended to replace the real world with a simulated environment [25–28]. The popularity of the AR system was expected to increase in the near future. The doctor could view images using a head-up or head-mounted display (such as HoloLens glasses), which enabled doctors to examine internal structures or lesions through covered tissue. AR also provides the physician with a visually sound anatomical structure [29–32]. Currently, virtual and real registration technology based on AR is a research hotspot. Lu et al. [33] designed a medical augmented reality system that locates the focal point by virtual and real registration technology and realized the application of virtual and real registration technology in brain surgery. Paloc et al. [34] discovered that virtual and real-time registration can be performed by magnetic markers, which promoted the computer-aided application of AR technology in liver surgery. AR technology has been employed for endoscopic navigation-assisted surgery, such as nasal and intestinal surgery, which achieved satisfactory results. First, models of organs and tissues were reconstructed by preoperative CT or MRI. Three-dimensional models of the patient’s brain tissue, eyeballs and blood vessels were then superimposed into the field of view in endoscopic surgery by virtual and real registration techniques. Zhuming et al. have achieved some breakthroughs in the study of human jaw bones using the virtual and real registration technology in AR. The virtual and real registration of the virtual jaw model and the real model has been completed [35]. In spinal surgery, the doctor had to obtain the patient’s three-dimensional bone structure. To achieve this goal, the patient needed to be photographed several times during the operation using a C-arm scanner from different positions. This process wasted the operation time and increased the risk of the patient being exposed to radiation. Essam et al. [36] proposed an AR imaging system for minimally invasive orthopaedic surgery. Augmented reality has a prominent role in the medical industry and a bright application prospect.

Based on the research on AR, this paper presented two methods to improve the accuracy of virtual and real registration. Virtual and real registration was carried out based on the spinal data obtained by CT. In the experimental scene of the operation, the real-time images of the spine were obtained. The accuracy of the AR navigation system was verified by virtual and real registration experiments.

## Results

In this study, with the help of orthopaedic surgeons, different groups of experiments were conducted to verify the efficacy of the proposed AR surgical navigation system.

### Accuracy of virtual and real registration based on general identification method

Four groups of experiments were designed to verify the accuracy of virtual and real registration based on the general identification method by changing the positions of markers on the spinal model. Specifically, the virtual model was overlapped with the real model by moving the logo. After the registration of virtual model and the real model was completed, the values of the coordinates of each marking point could be read and recorded in real time. The real model reappeared in the video stream. The position of each of the small balls fixed on the model was read the by Northern Digital Inc (NDI) optical tracking system. The values of their coordinates in the world coordinate system were calculated. The registration error of each point could be calculated by comparing the previously recorded coordinate values. The average error of each group was calculated by the registration error of the points obtained from each group of experiments. The experimental results are listed in Table 1.

**Table 1:** Error of virtual and real registration based on identification method (mm)

Group	Point	Mark points on the real model			Mark points on the virtual model			Error (mm)	Average error (mm)
		*X*	*Y*	*Z*	*X*	*Y*	*Z*		
A	P1	45.70	− 25.95	− 1363.48	55.87	− 28.01	− 1361.26	10.61	10.23
	P2	47.81	− 0.78	− 1323.44	54.52	− 6.91	− 1324.59	9.16	
	P3	58.86	48.77	− 1327.13	51.42	46.80	− 1334.29	10.51	
	P4	69.86	− 24.88	− 1332.28	66.00	− 20.89	− 1341.33	10.62	
B	P1	165.24	− 108.11	− 1206.25	160.57	− 104.36	− 1208.38	9.71	9.98
	P2	151.02	− 72.56	− 1214.28	156.34	− 69.75	− 1210.33	10.02	
	P3	135.46	− 28.12	− 1216.35	141.28	− 27.34	− 1222.28	9.86	
	P4	198.23	− 93.28	− 1208.21	191.29	− 91.34	− 1200.05	10.36	
C	P1	− 144.83	− 220.59	− 1500.30	− 139.32	− 225.31	− 1507.44	10.15	8.99
	P2	− 143.56	− 219.11	− 1453.06	− 138.04	− 215.58	− 1458.95	8.82	
	P3	− 111.46	− 186.21	− 1431.23	− 106.91	− 190.55	− 1436.52	8.23	
	P4	− 132.74	− 245.08	− 1471.85	− 127.87	− 248.28	− 1478.40	8.77	
D	P1	196.32	− 86.45	− 1220.78	189.36	− 89.25	− 1214.33	10.06	9.70
	P2	169.42	− 48.69	− 1215.68	162.98	− 50.21	− 1209.22	10.02	
	P3	162.94	− 21.69	− 1250.64	170.75	− 23.12	− 1145.88	9.26	
	P4	190.32	− 58.96	− 1178.14	182.45	− 54.31	− 1196.34	9.45	

As shown in the experimental data in Table 1, the average accuracy of the virtual and real registration experiment was 9.73 ± 0.46 mm (range 8.90–10.23 mm). The difference between the upper limit and lower limit was approximately 2.39 mm, and the distribution of the experimental results was scattered. The results concluded that the accuracy of the virtual and real registration based on the general identification method was low and the stability was poor. Achieving the high-precision registration effect was not feasible. The method of manually adjusting the logo has been employed throughout the experiment, which was often difficult and not practical in the course of actual operation.

### Accuracy of virtual and real registration based on improved identification method

Virtual and real registration-based general identification method has some problems, such as low accuracy and stability. To solve these problems, control of the logo by software to achieve secondary registration was introduced. In the registration process, the hand-held logo could be used to move the virtual model to the position of the real model in the space to achieve the first registration. The second registration was carried out by using the keyboard input to move or rotate the virtual model. The virtual and real registration experiments of four groups of different points were carried out by changing the locations of the markers. The experimental results of the virtual and real registration obtained by the improved identification method are shown in Table 2.

**Table 2:** Error of virtual and real registration based on improved identification method (mm)

Group	Point	Mark points on the real model			Mark points on the virtual model			Coordinate error			Error (mm)	Average error (mm)	*p*-value
		*X*	*Y*	*Z*	*X*	*Y*	*Z*	*X*	*Y*	*Z*			
A	P1	− 138.41	− 153.00	− 1381.74	− 139.57	− 152.82	− 1384.68	1.16	0.18	2.93	3.16	3.52	0.65
	P2	− 152.42	− 72.01	− 1377.30	− 149.08	− 69.94	− 1376.99	3.35	2.07	0.31	3.95		
	P3	− 120.67	− 134.73	− 1349.59	− 123.99	− 133.29	− 1351.58	3.32	1.44	1.98	4.13		
	P4	− 175.34	− 81.00	− 1424.07	− 176.85	− 80.06	− 1424.46	1.52	0.40	2.40	2.86		
B	P1	− 62.10	185.31	− 1390.53	− 64.94	184.02	− 1390.24	2.84	1.29	0.29	3.13	3.36	
	P2	− 28.07	149.31	− 1328.60	− 30.94	148.22	− 1328.02	2.87	1.09	0.58	3.20		
	P3	− 0.91	202.92	− 1280.62	− 1.94	201.79	− 1277.72	1.03	1.13	2.90	3.28		
	P4	− 84.33	191.37	− 1424.81	− 80.02	189.98	− 1424.76	4.31	1.39	0.05	3.83		
C	P1	− 37.84	186.61	− 1352.84	− 40.17	− 184.76	− 1352.93	2.33	1.85	0.09	2.97	3.53	0.60
	P2	− 36.07	178.86	− 1307.83	− 38.87	176.72	− 1307.93	2.80	2.14	0.10	3.52		
	P3	− 65.96	259.36	− 1381.28	− 67.42	258.92	− 1384.73	1.46	0.44	3.45	3.77		
	P4	− 108.21	− 63.52	− 1210.20	− 110.43	− 60.58	− 1211.05	2.22	2.94	0.85	3.84		
D	P1	− 87.14	119.06	− 1316.18	− 84.64	121.76	− 1317.87	2.50	2.70	1.69	4.32	3.73	
	P2	− 77.20	112.31	− 1270.39	− 74.86	108.97	− 1268.34	2.34	3.34	2.05	4.28		
	P3	− 39.13	139.35	− 1250.14	− 40.71	141.02	− 1246.47	1.58	1.67	3.67	3.13		
	P4	− 72.09	89.47	− 1294.83	− 70.64	91.45	− 1291.38	1.45	1.98	3.45	3.21		

As shown in Table 2, the average accuracy of the experiment based on the improved identification method was 3.54 ± 0.13 mm (range 3.36–3.73 mm), and the distribution of experimental data was concentrated. The maximum value of the virtual and real registration accuracy of a single point was 4.32 mm. To observe the error of the virtual and real registration of each point more clearly, calculation of the error of the *X*, *Y* and *Z* coordinates was added. According to the experimental data in the table, the accuracy of the virtual and real registration based on the improved identification method has been significantly improved, and the stability was enhanced.

### Accuracy of virtual and real registration based on robot-assisted method

Based on the virtual and real registration method of the improved identification method, the robot was introduced instead of manual puncture to address the problem of human error in the puncture process. The experimental data obtained by the four groups of experiments are shown in Table 3.

**Table 3:** Error of virtual and real registration based on robot-assisted method (mm)

Group	Mark point on the real model			Mark point on the virtual model			Coordinate error			Error (mm)	Average error (mm)	*p*-value
	*X*	*Y*	*Z*	*X*	*Y*	*Z*	*X*	*Y*	*Z*			
A	− 40.23	174.56	− 1307.03	− 38.16	174.90	− 1305.77	2.07	0.34	1.26	2.45	2.39	0.17
				− 38.63	175.67	− 1305.53	1.60	1.11	1.50	2.46		
				− 38.75	175.77	− 1305.92	1.48	1.21	1.11	2.21		
				− 39.14	176.61	− 1306.33	1.09	2.05	0.70	2.42		
B	− 45.69	192.38	− 1346.24	− 44.97	191.34	− 1345.01	0.72	1.04	1.23	2.36	2.52	
				− 47.02	190.53	− 1345.15	1.33	1.85	1.09	2.53		
				− 44.54	194.09	− 1347.67	1.15	1.71	1.43	2.51		
				− 47.43	190.73	− 1347.45	1.74	1.65	1.21	2.69		
C	− 1.91	202.92	− 1280.62	− 2.98	204.28	− 1278.89	1.07	1.36	1.73	2.41	2.55	0.76
				− 3.27	203.55	− 1278.36	1.36	0.63	2.26	2.71		
				− 2.94	201.79	− 1278.72	1.03	1.13	1.90	2.44		
				− 3.23	202.32	− 1282.79	1.32	0.60	2.17	2.61		
D	− 26.07	149.31	− 1328.60	− 25.15	151.39	− 1328.22	0.92	2.08	0.38	2.31	2.58	
				− 27.53	147.45	− 1330.07	1.46	1.86	1.47	2.78		
				− 24.63	147.92	− 1330.41	1.44	1.39	1.81	2.57		
				− 26.65	146.86	− 1328.99	0.58	2.45	0.39	2.66		

As shown in the experimental data of the robot puncture, the total experimental accuracy has been further improved based on the improved identification method. The average accuracy of four groups of virtual and real registration experiments was 2.51 ± 0.07 mm (range 2.39–2.58 mm), and the accuracy of single point registration was approximately 2.5 mm.

### Statistical analysis of the results of experiments

The experiments were expected to achieve accuracy within 3.5 mm. To facilitate the summary of the data in Tables 1, 2 and 3, the point where the accuracy was 0–2.5 mm was defined as grade A. The accuracy of 2.5–3.5 mm, 3.5–4.5 mm and more than 4.5 mm were defined as grade B, grade C and grade D, respectively. The accuracy of grade A and grade B was regarded as the best accuracy and acceptable accuracy, respectively. The accuracy of grades C and D was regarded as the deviation in the greater precision and the meaningless precision, as shown in Table 4.

**Table 4:** Statistical analysis of experimental results

Methods	Range of error (mm)	Grade	Number of points
Robot experiments	0–2.50	A	8
	2.50–3.50	B	8
	3.50–4.50	C	0
Improved identification method	0–2.50	A	0
	2.50–3.50	B	8
	3.5–4.50	C	8
Identification method	≤ 4.50	C	0
	> 4.50	D	16

#### Experimental data

SPSS Statistics Version 25 software (IBM, Armonk, NY, USA) was employed for the statistical analysis. The data were expressed as the mean ± standard deviation. The TTEST accurate test was adopted to determine whether a significant relationship existed between each group of data. A *p*-value analysis of virtual and real registration errors based on the robot-assisted method was presented. The *p*-value between group A and group B was 0.17 (if the *p*-value is > 0.05, the data of the two groups are not significantly different). The results concluded that no significant difference exists between the data of group A and group B. The *p*-value between group C and group D was 0.76. No significant difference exists between the data of group C and the data of group D. The *p*-value of the virtual and real registration based on the improved identification method was calculated. The *p*-value between group A and group B was 0.65, and the *p*-value between group C and group D was 0.60. The *p*-value of the experimental data reveals no significant difference between each group of data of the same experimental method.

From the analysis of the statistical results, the accuracy of the virtual and real registration experiments of the 16 points that was based on the general identification method were large, which exceeds the acceptable range. In the actual experimental process, registration with the actual model by moving the logo was more difficult. If the hand-held logo was slightly shaken, it would cause a large error in the registration, which will directly cause the experiments to fail. Based on the quadratic registration of the improved identification method, the accuracy of the virtual and real registration of the 16 points considerably improved. The accuracy of the virtual and real registration was 8 points in grade B, which accounts for 50% of the total number of points. According to the total experimental results, the number of points that achieve at least grade C or above was 16, which is 100% higher than the previous general identification method. According to the experimental results, however, the number of points that achieve grade A was 0, while the number of points that exceed grade B accounted for 50% of the total. This method remained problematic, for example, the error of manual puncture and the single visual angle of human eye. After using the robot puncture, a distinct effect has been obtained. The number of points that achieve grade A accounted for 50% of the total, and the remaining points were within the acceptable range.

**Fig. 1:**
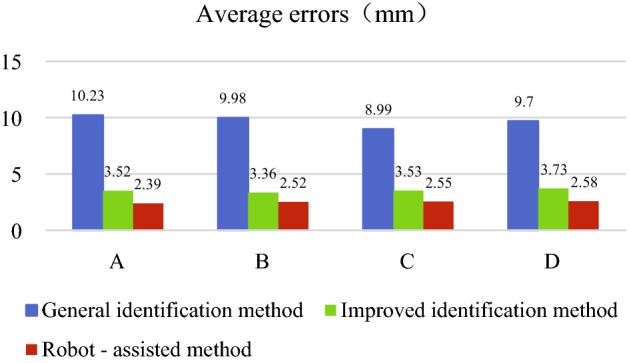
Comparison of the average error of virtual and real registration in four groups of three methods

As shown in Fig. 1, based on the general identification method, two optimizations have achieved satisfactory results. Based on four groups of different experimental results, the accuracy of the virtual and real registration from the general identification method to the improved identification method has been improved most significantly, from approximately 9.8 mm to approximately 3.5 mm, with an increase of 65%. The comparison of the accuracy of the virtual and real registration between the two methods directly indicated the feasibility and advantages of the method based on the improved identification method. Based on the improved identification method, a robot was introduced to perform the puncture. The average error of the virtual and real registration experiments decreased from approximately 3.5 mm to approximately 2.5 mm, and the accuracy of the entire virtual and real registration increased by approximately 28.5%. The average accuracy of the other two methods was relatively stable.

**Fig. 2:**
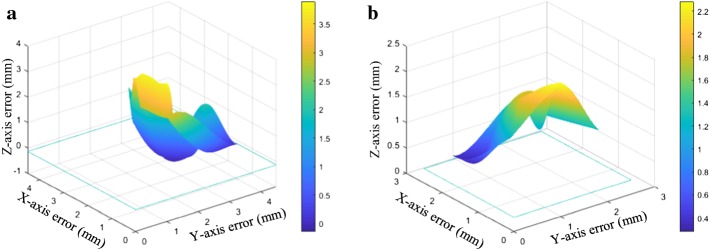
Errors in *X*, *Y* and Z directions based on improved identification method and robot-assisted method. **a** The error in the *x*, *y* and *z* directions based on improved identification method; **b** the error in the *x*, *y* and z directions based on improved robot-assisted method

In the process of the virtual and real registration experiment based on the improved identification method and robot-assisted method, the errors of the *X*, *Y* and *Z* coordinates were analysed. Figure 2 shows the distribution of errors in the coordinates of the *X*, *Y* and *Z* axes of each point. As shown in Fig. 2a, the errors in the direction of each axis of the virtual and real registration based on the improved identification method. As shown in Fig. 2b, the errors of the *X*, *Y* and *Z* axes were concentrated between 0.5 and 2 mm, and the images were relatively compact. Approximately 60% of the error area was concentrated between 0.5 and 2.5 mm, and the remainder of the error area was distributed between 2.5 and 3.5 mm. The image hierarchy of the errors of the *X*, *Y* and *Z* axes based on the robot-assisted method was relatively distinct, approximately 30% in the blue region, and the error range was 0.4–1 mm. A total of approximately 20% of the errors were in the green area, and the remaining errors were in the yellow area. Compared with the improved identification method, the error of the robot-assisted method in each coordinate direction was considerably smaller, which indirectly indicated that the robot-assisted method has higher accuracy and stability. The errors of the coordinates of the *X*, *Y* and *Z* axes were irregular.

**Fig. 3:**
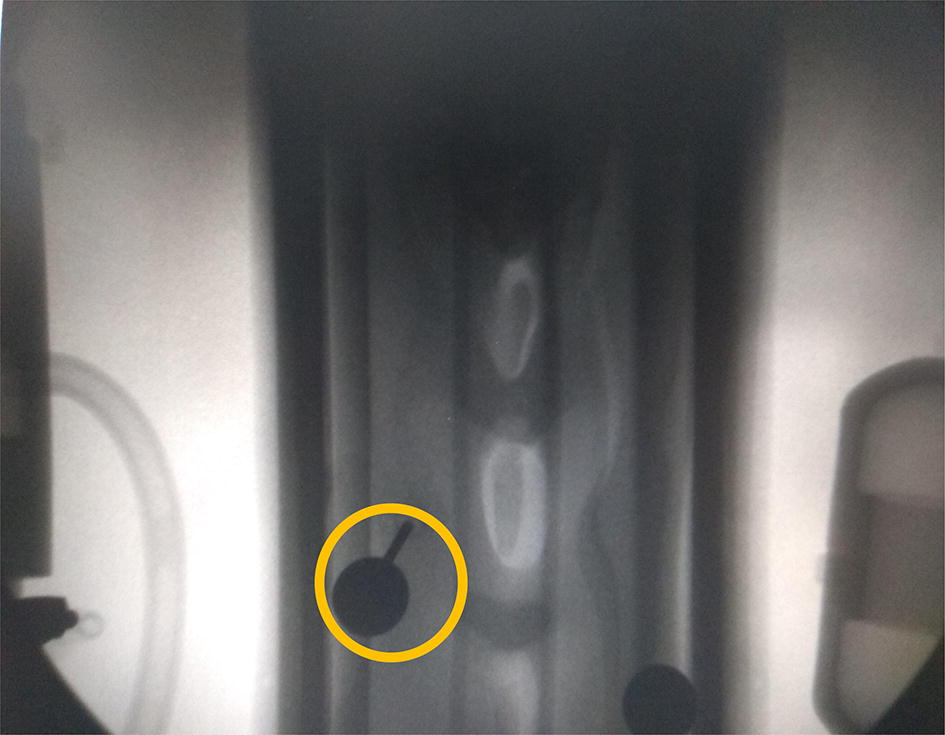
Verification of the effect of the robot puncture mark point by X-ray image

In order to verify that the robot can replace the human hand to pick up the marker points on the virtual model, the probe on the robot end-effector was inserted into the position of the virtual target marker and remains stationary. As shown in Fig. 3, X-ray images showed that the tip of the probe on the end-effector of the robot was located in the center of the robot in two puncture and point-taking experiments in different positions. The discovery suggests that robots could completely replace the person who is going through the puncture.

## Discussion

In this study, robot-assisted surgery was introduced to develop an AR surgical navigation system based on an improved identification method for intraoperative spinal puncture. The developed AR navigation system could accurately overlay the image of the 3D spine onto the model of the spine. The system has the advantages of no radiation and satisfactory anti-interference.

### Comparison of spine surgery without robotic assistance based on AR

As shown in Table 5, some progress had been made in the efforts to develop an AR surgical navigation system. The research methods adopted in this study differ from other studies. This study uses software to freely adjust the logo, which has strong adaptability and flexibility. The accuracy of this study is higher than the accuracy of other studies, and the stability of the system is excellent. As early as 2016, Kleck et al. [37] employed the O-arm and StealthStation to evaluate the accuracy of the three-dimensional navigation of the pedicle screw. The obtained navigation accuracy is approximately 5.9 ± 3.5 mm. Although the 2D to 3D surgical navigation has been realized, it has a large deviation from the actual application. In the same year, Guha et al. [38] validated clinical pedicle implantation based on the correlation between postoperative imaging and absolute quantitative navigation accuracy. These researchers achieved a high precision of approximately 1.8 mm. Exhibiting an up-and-down deviation of 3.6 mm, the instability of the navigation system is indirectly explained. In contrast, the navigation system that we investigated is less accurate, but our navigation system is superior in stability. The development of entry-point guidance prior to vertebroplasty spinal surgery is an important issue. In 2017, Wu et al. developed an advanced AR system for assisting spinal surgery [39]. The research has a certain breakthrough significance, but the research results are between 1.4 and 12.3 mm, with very large upper and lower limits of error. While a certain research prospect is proposed for the future, no satisfactory result has been obtained.

**Table 5:** Summary of articles that report technical accuracy from augmented reality navigation system

Year-author [References]	Technical accuracy (mm)	Main findings
2016-Kleck et al. [37]	5.9 ± 3.5	Description of a novel method for evaluation of pedicle screws in 3 dimensions utilizing O-arm and StealthStation navigation
2016-Guha et al. [38]	1.8 ± 3.6	To characterize the correlation between clinical pedicle screw accuracy, based on postoperative imaging, and absolute quantitative navigation accuracy
2017-Wu et al. [39]	1.4–12.3	Present an advanced AR system for spinal surgery assistance, and develops entry-point guidance prior to vertebroplasty spinal surgery
2019-Müller et al. [40]	3.4 ± 1.6	An intraoperative 3D imaging AR navigation system for pedicle screw fixation was developed
2019-Urakov et al. [41]	1–5	Discusses the potential and limitations of AR in its current state and identifies strategies for successful AR application in future surgery
This study	2.5 ± 0.5	The software method is used to improve the marking method to achieve higher puncture precision. The robot-assisted surgical puncture that may be realized in the future has been verified and good results have been obtained

In 2019, Fabio Muller et al. developed an intraoperative 3D imaging AR navigation system for pedicle screw internal fixation. During the operation, the preoperative plan was registered via three-dimensional fluoroscopy and the reference mark on the lumbar spine, and the customized drill sleeve guide rail can be tracked to achieve real-time navigation. The average translational error of the final navigation was 3.4 ± 1.6 mm. This study also faces the limitations of using HMD for AR navigation. Simultaneously tracking two or more markers as the HMD moves through space can sometimes cause the hologram to wobble and may even require a reboot. Compared with our study, the accuracy and stability proposed by this study are slightly lower than those of our system [40]. In response to the analysis of the future development of AR navigation systems, Urakov et al. discussed the potential and limitations of AR in the current state in 2019 [41]. AR will be successfully applied in future surgeries, with an accuracy as low as 1 mm.

In comparison with Table 6, the accuracy of robot-assisted spine surgery was significantly higher than that of freehand operation. In terms of radiation exposure, robot-assisted spine surgery took less time than freehand operation.

**Table 6:** Analysis of robot-assisted spinal surgery based on AR

Authors and year	Surgical approach	Radiation exposure time (s/screw or puncture)	Study type	Accuracy (%)	Complications
Kantelhardt et al. 2011	Open and percutaneous	Freehand: 77.0; robotic-guided open: 43.0	Retrospective	Freehand: 91.4; robotic: 94.5	8
Keric et al. 2017	Percutaneous	22.4	Retrospective	96.9	13
Hyun et al. 2016	Open and percutaneous	Freehand: 13.3; robotic: 3.5	Prospective	Freehand: 98.6; robotic: 100	2
Lonjon et al. 2015	Open	Freehand: 4.8; robotic: 18.5	Prospective	Freehand: 92; robotic: 97.3	3
Kim et al. 2016	Open and percutaneous	4.8	Prospective	Freehand:99.4; robotic:99.4	None
Roser et al. 2013	Open and percutaneous	Freehand: 31.5; robotic: 16.0	Prospective	Freehand: 97.5; robotic: 99	None
Solomiichuk et al. 2017	Open and percutaneous	Freehand: 20.7; robotic: 25.1	Retrospective	Freehand: 83.6; robotic: 84.4	None
Macke et al. 2016	Open	Not listed	Retrospective	92.8; 97.6	None
This study	Minimally invasive	Freehand: 2; robotic: 2	Prospective	Freehand: 50; robotic: 100	None

### Comparison of robot-assisted spine surgery based on AR

#### Surgical approach

Open surgery was discussed in all seven studies listed in the comparative literature. In our study, robot-assisted minimally invasive surgery based on AR technology was adopted. Compared with open surgery, minimally invasive surgery has the advantages of smaller incisions and less harm to the patient. In the future, minimally invasive surgery will probably become the mainstream in the selection of spinal surgery.

#### Radiation exposure time

Eight studies evaluated radiation exposure to the surgical team (Table 6). Radiation exposure time (RET) determination for each screw placement or surgical instrument puncture requires a few seconds of fluorescent examination to ensure consistency. In a study by Lonjon et al. [42], the average RET for bare-handed operations was 4.8 s/screw, while the average RET for ROSA operations was 18.5 s/screw. Kantelhardt et al. [43] have made an in-depth contrast between robot-assisted open and percutaneous surgery and traditional freehand operations. The average RET of conventional surgery was 77 s/screw, while the average RET of robot-guided open surgery was 43 s/screw, and the average RET of robot-guided percutaneous surgery was 27 s/screw. Keric et al. [44] analysed the results of 90 patients and discovered that the average RETs for bare-handed surgery were longer than those for robot-assisted surgery, at 56.4 s/screw and 24 s/screw, respectively. They attributed the increased radiation time to their practice of using only 2 images (AP and lateral) to accurately match ROSA’s images while they applied 5.3 images per patient. In our study, no radiation existed during the operation as no auxiliary means such as X-ray were used to observe the puncture of surgical instruments. The amount and time of radiation obtained remained constant regardless of the adoption of manual operation or robot assistance. The amount of radiation in this study was only obtained from examination of the puncture effect after the puncture of the surgical instrument; thus, it had less radiation time than other studies.

#### Accuracy

The accuracy of robot-assisted surgery listed in this paper is shown in Table 6. Roser et al. [45] investigated patients with lumbar spine instability and discovered that the accuracy of the freehand technique was 97.5% and the accuracy of the spinal assistance tool was 99%. Hyun et al. [46] compared the accuracy rate of the robot group with the robot-assisted percutaneous surgery and determined that the accuracy rate of the robot group was 100%, while the accuracy rate of the freehand group was 98.6%. Lonjon et al. revealed that the Rosa robot-assisted screw placement was accurate 97.3% of the time, compared with 92% for the freehand group. Based on Spine Assist’s research, Solomiichuk et al. [47] showed that the accuracy rate of freehand was 83.6%, while that of the robot was only 84.4%. The difference between the two rates was not significant. The study suggests that one possible explanation for the reduced accuracy of robot-assisted surgery is the lack of available spinal contours on which robots rely for image recognition and segmentation.

Macke et al. explored adolescent idiopathic scoliosis patients. During surgery, screw placement was difficult as the pedicle was shorter in the child [48]. The final results showed that the accuracy of robotic assistance ranged from 92.8 to 97.6%. The results showed that prone imaging was more accurate. Postoperative CT scan images were used to measure the accuracy of screw placement. Keric et al. observed differences in the success rates between robot-assisted surgery and open surgery (90% vs 73.5%) and attributed this difference to whether better trajectory planning could be achieved with the assistance of preoperative robots, which suggests that preoperative trajectory planning had substantial value for robotic surgical navigation. In a study by Kim et al. [49], the contact pressure between facet joints and intervertebral discs in the robot group was significantly lower than those in the open freehand group.

In our study, the accuracy of grade A and grade B was regarded the best accuracy and acceptable accuracy, respectively. Four groups of 16 experiments were designed for robot-assisted puncture and freehand operation. The results showed that the qualified rate of experimental accuracy of freehand puncture was approximately 50%. The maximum accuracy of robot-assisted puncture was 100%. The puncture accuracy based on robot-assisted surgery has a considerable advantage over the freehand operation.

As shown in Table 6, the maximum accuracy of most robot-assisted surgeries was 90%. Due to the lack of available spine contour, the robot relies on the spine contour for image recognition and segmentation. The accuracy of robot-assisted surgery proposed by Solomiichuk et al. was only 84.4%. The precision of the study by Hyun et al. and the robot-assisted surgery explored in our paper can reach 100%. Therefore, the finding indirectly shows that robot-assisted surgery can optimize and improve the accuracy of the surgical navigation system in this study to a certain extent.

The accuracy of the robot’s puncture accuracy seemed to be within an acceptable standard of care. However, the results of the comparison between robotic technology and traditional surgical treatment in terms of surgical duration and other indicators remain uncertain. Proving the rationality of the extensive application is difficult. Future research including research by surgeons with extensive robotic experience, beyond the recommended learning curve is needed.

### Preoperative or intraoperative imaging

Images of lesion points in patients during and before surgery can be obtained using many ways, and different ways had different effects on the operation. In most cases, preoperative images of the patient were obtained by CT or MRI [50, 51]. However, images of the patient’s focal point during an operation can be obtained using different ways. Hooman et al. evaluated the location of pedicle screw fixation using 2D–3D registration of preoperative computed tomography (CT) and intraoperative projection images (X-rays) [52]. The feasibility of the rigid-body-based 2D–3D registration method described in this paper was demonstrated. However, continuous intraoperative X-ray images of the patient’s focal points can expose both the physician and the patient to large amounts of radiation, which does not satisfy the doctor’s need for spine surgery. Zhang et al. [53] applied image reconstruction based on three-dimensional models to clinical studies. The image quality was improved by incorporating surgical instrument models (“known components”) in the joint image register–reconstruction process. The O‐arm system for CBCT was intraoperatively deployed to obtain image information of the patient’s spine. Although the algorithm’s potential low-dose advantage was tested by simulating low-dose data in images obtained at normal doses (as low as one-tenth of the standard protocol dose), compared with our study, the effect of radiation remains. The navigation system that we evaluated based on AR for spinal surgery does not present any radiation problems during surgery. In our study, a high-definition camera was used to capture the surgery scene in real time and transmit it to a computer. The video signal after virtual and real registration was obtained by the computer and then output in real time by a 3D display. During the operation, real-time registration of three-dimensional medical images and the surgical site was realized. Doctors can view the structure of the spine in real time through the patient’s skin.

### Method of intraoperative tracking

By connecting depth data to robot-assisted navigation, the AR navigation system proposed by He et al. [54] can automatically track and avoid obstacles that may block the femur. Instead of using optical markers, the study’s registration system was built on a depth camera using robotics. The end-effector of the serial manipulator is captured by a depth camera. A depth camera was used to dynamically track the target bone during the process. The cone area is defined according to the line between the target and the camera, and the objects inside the cone detected by the depth camera are tracked as obstacles. This method can easily cause loss of target tracking. Ma et al. [55] proposed a remote IMN-interlocked AR surgical navigation method that is based on the combination of optical and electromagnetic tracking. Two optical markers were attached to the drill and IV stack for optical tracking. An optical marker for hybrid positioning was fixed on an electromagnetic launcher. Intraoperatively, an optical tracking driller and a vein covering device were employed, and IMN electromagnetic tracking was applied. The hybrid photoelectric tracking method is more accurate and reliable than separately using the two systems. However, the ferromagnetic material of the surgical instrument would cause electromagnetic field distortion; thus, only the non-magnetic material of the surgical instrument could be applied. In addition, the sensor is not encapsulated in sterilizable non-ferrous metal tubes, which may affect the operation. Compared with these two methods, our study employed an optical tracking system to track patients and surgical instruments in real time during surgery. The intraoperative tracking was established by using the optical tracking system to obtain information about the surgical instrument and the optical marker of the patient’s lesion in real time. Compared with other tracking methods, the optical tracking system has higher precision and real-time performance. No special requirements are needed for the materials and appearance of surgical instruments.

### Display device in surgical navigation based on AR

Currently, the commonly employed interactive devices in AR technology were the head-mounted display (HMD), enhanced lens and enhanced display. Head-mounted displays were the most frequently employed displays in AR systems [56–58]. Gibby et al. [59] tested pedicle screw placement without real-time fluoroscopy by head-mounted display of augmented reality (HMD-AR) and superimposed computed tomography (CT) data. Compared with the data of percutaneous pedicle screw placement without HMD-AR, the operation time was shortened and the accuracy of pedicle insertion was improved. The device can only be operated by voice commands or gestures; thus, the surgeon’s hands remain free and sterile throughout the operation. However, the head-mounted display was susceptible to the influence of the surrounding environment, which caused the deviation in the registration results or a poor display effect. In addition, most surgeries were complicated and required a long time. If the doctor wore the head-mounted display for a long time, it would cause discomfort to the doctor and affect the operation. Carl et al. [60] applied the method of AR operation microscopy to spine surgery. The sectional structure of the surgical area can be visually displayed by the upside-down display of the operating microscope. The video of the microscope was superimposed with the segmented 3D structure, and the segmented 3D structure was visualized in a semi-transparent way with various display methods of image data. The study has limitations and was only preliminary. In the process of surgery, the operation of the operating microscope was more complicated. The equipment has low integration and AR calibration was relatively difficult. In future research, the equipment should be integrated into a hand-held device that is similar to a tablet. The enhanced display was a method of fusion display in a 3D display after registration of a virtual 3D model and real surgical scene. Our study chose to use enhanced displays. Compared with other display devices, this interactive method can separate the complex computing module from the display module, which ensured that the entire system had the characteristics of low coupling and could be subsequently maintained and expanded. A helmet or glasses was not required, and an extra AR device between the doctor and the patient was not necessary, which rendered the operation neither difficult nor uncomfortable. During the operation, doctors can view the anatomical structure and surgical instruments of patients in the real surgical area at any time by a 3D display, which can reduce the difficulty of the operation and increase the efficiency and success rate of the operation.

### Limitations

In our study, virtual and real registration was performed based on the rigid body registration principle, to realize the function of AR surgical navigation. Due to slight changes in the patient’s breathing or posture during the actual process, the registration between the model image and the patient’s spine may be complicated. Our study did not take this problem into account. However, if we want to further improve the registration accuracy and enhance the practical applications of this system, this problem must be overcome. Due to the limited perspective of human eyes, the virtual model and the real model may appear to have been completely matched in a certain perspective during the experiment. If the perspective were changed, some parts would not overlap well. This deficiency undoubtedly increases the error of virtual and real registration, which directly affects the precision of surgical navigation. If multiple cameras were introduced into the study for multi-view registration from different angles, the accuracy of virtual and real registration could be further improved.

## Conclusions

In this paper, the 3D reconstruction of the spinal model was superimposed in a real scene by building a platform of surgical navigation based on AR. Subsequently, experiments were carried out to identify the virtual and real registration of the spinal model by using the identification method. In addition, the function of adjusting the virtual model was added based on registration that was based on the general identification method. Secondary registration was employed to increase the accuracy of registration and reduce the shortcomings of registration based on the general identification method. The method of robot puncture could reduce the error of human operation. To verify the accuracy of VR registration, an experiment and error calculation were carried out.

The final experimental results show that the highest accuracy of virtual and real registration based on the robot-assisted method was 2.39 mm. The average accuracy of virtual and real registration based on the general identification method was 9.73 ± 0.46 mm (range 8.90–10.23 mm). Compared with the virtual and real registration based on the general identification method, the accuracy was improved by approximately 75%. Therefore, the experimental results showed that the two optimized methods were highly effective. During the entire experiments, the virtual spinal model obtained from 3D reconstruction could be matched with the real spinal model via the AR navigation system. Thus, the location and structure information of the spinal lesion could be directly observed.

## Methods

### AR navigation system design

#### Hardware composition of the system platform

**Fig. 4:**
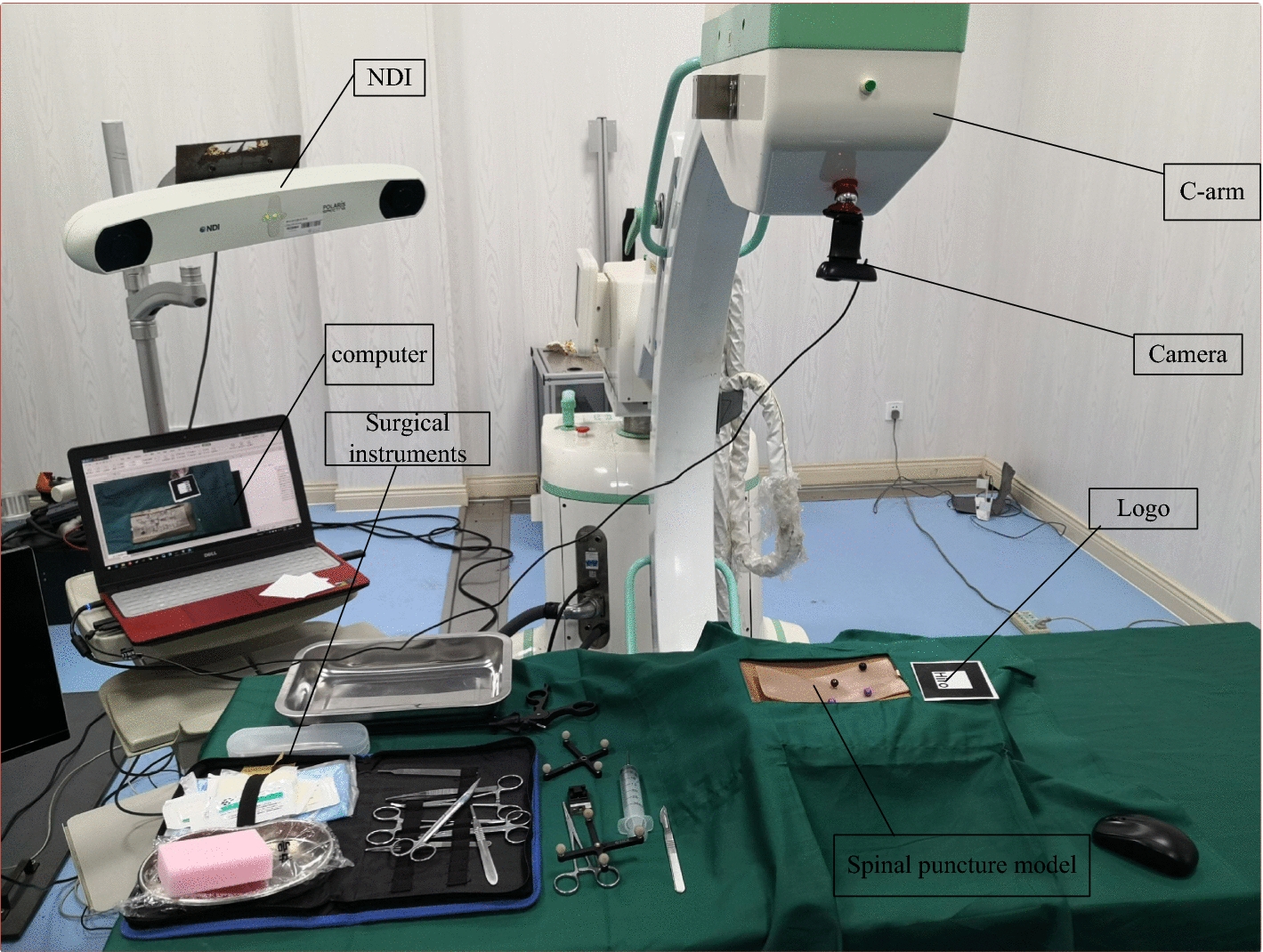
Hardware composition of the system

**Fig. 5:**
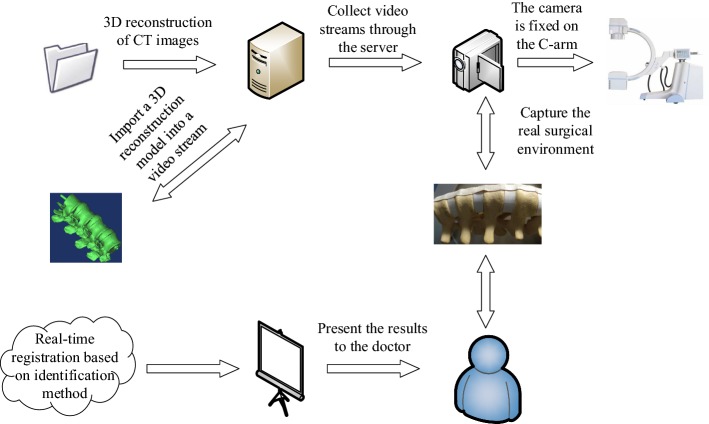
Composition and function of the hardware of the system

The hardware of the system was composed as shown in Fig. 4. The experimental system consisted of a computer, a camera, experimental platform, spinal model, a logo, surgical instruments, NDI optical tracking system and C-arm. The camera was fixed on the C-arm throughout the experiment. The angle and height could be adjusted by the rotation of the C-arm to provide a better position [61, 62]. The functions of the hardware in the system are shown in Fig. 5.

#### Camera calibration

**Fig. 6:**
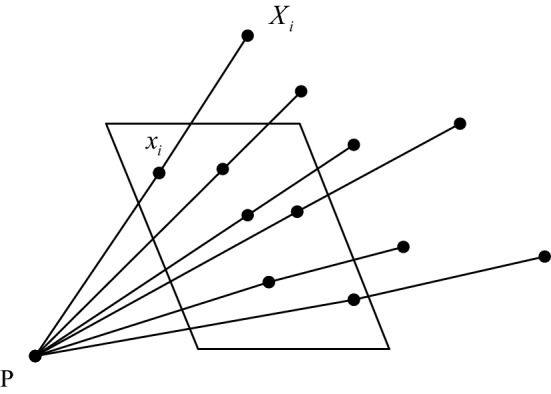
Camera calibration diagram

The main purpose of camera calibration was to calculate the camera’s internal parameters, external parameters and distortion parameters [63]. The process of camera calibration, which is shown in Fig. 6, was to obtain the 3D point $X_{i}$ of the world coordinates and the 2D point $x_{i}$ of the image coordinates. The transformation of these 3D points to 2D points could be obtained by a series of matrix transformations.

**Fig. 7:**
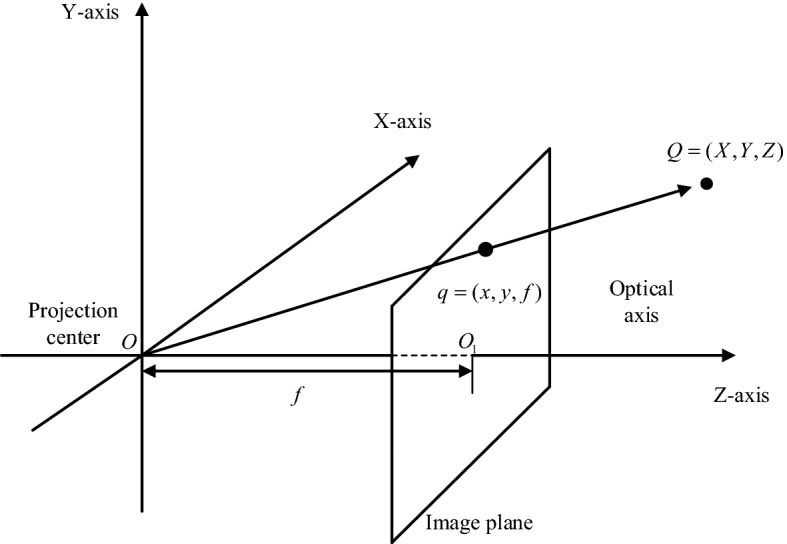
Camera model

The entire calibration process was divided into two parts. The first step was to convert from the world coordinate system to the camera coordinate system. This step was the transformation from 3D point to 3D point, including *R*, *t* and other parameters to determine the location and orientation of the camera in 3D space. The second step was to convert from a camera coordinate system to a retinal coordinate system. This step was the transformation from 3D point to 2D point, including the internal parameter *K* of the camera. The model of the camera is shown in Fig. 7. The *O* point represented the centre point of the camera and was also the centre point of the camera coordinate system. The *z*-axis was the main axis of the camera. The point *O*_1_ represented the intersection of the main axis and the image plane. The distance from *O* to *O*_1_ was the focal length of the camera. The pixel coordinate system and the retinal coordinate system were on the same plane, with the exception that the origin was different.

The conversion relationship between the world coordinate system and the camera coordinate system could be obtained by using the rotation matrix *R* and the translation matrix *t*:

1 $$\left[ \begin{aligned} X_{\text{C}} \\ Y_{\text{C}} \\ Z_{\text{C}} \\ 1 \\ \end{aligned} \right] = \left[ {\begin{array}{*{20}c} R & t \\ {0^{\text{T}} } & 1 \\ \end{array} } \right]\left[ \begin{aligned} X \\ Y \\ Z \\ 1 \\ \end{aligned} \right] = T_{\text{CW}} \left[ \begin{aligned} X \\ Y \\ Z \\ 1 \\ \end{aligned} \right].$$

*R* was the rotation matrix about three coordinate axes. *T* was referred to as the three-dimensional translation vector, which was used to represent the relative pose between the world coordinate system and the camera coordinate system. $(X_{\text{C}} ,Y_{\text{C}} ,Z_{\text{C}} ,1)^{\text{T}}$ represented the coordinate of point $(X,Y,Z,1)^{\text{T}}$ in the camera coordinate system. $T_{\text{CW}}$ was an external parameter matrix of the camera composed of the rotation matrix *R* and the translation vector *t*, which represented a conversion relationship between the world coordinate system and the camera coordinate system.

The conversion relationship between the camera coordinate system and the retinal coordinate system is expressed as:

2 $$Z_{\text{C}} \left[ \begin{aligned} x \\ y \\ 1 \\ \end{aligned} \right] = \left[ {\begin{array}{*{20}c} f & 0 & 0 & 0 \\ 0 & f & 0 & 0 \\ 0 & 0 & 1 & 0 \\ \end{array} } \right]\left[ \begin{aligned} X_{\text{C}} \\ Y_{\text{C}} \\ Z_{\text{C}} \\ 1 \\ \end{aligned} \right],$$

where $\left( {x,y,1} \right)^{\text{T}}$ was the coordinate of the imaging point in the retinal coordinate system.

The conversion relationship between the retinal coordinate system and the pixel coordinate system:

3 $$\left[ \begin{aligned} u \\ v \\ 1 \\ \end{aligned} \right] = \left[ {\begin{array}{*{20}c} {\frac{1}{{d_{x} }}} & 0 & {u_{0} } \\ 0 & {\frac{1}{{d_{y} }}} & {v_{0} } \\ 0 & 0 & 0 \\ \end{array} } \right]\left[ \begin{aligned} x \\ y \\ 1 \\ \end{aligned} \right].$$

In the formula, $\left( {u,v} \right)$ represented the coordinates of the imaged point in the retinal coordinate system. $\left( {u_{0} ,v_{0} } \right)$ was the coordinate of the main point of the camera in the retinal coordinate system. $d_{x} ,d_{v}$ represented the physical dimensions along the *x*-axes and *y*-axes of each pixel in the image coordinate system. These variables could be obtained by formulae 1, 2 and 3:

4 $$Z_{\text{C}} \left[ \begin{aligned} u \\ v \\ 1 \\ \end{aligned} \right] = \left[ {\begin{array}{*{20}c} {\frac{1}{{d_{x} }}} & 0 & {u_{0} } \\ 0 & {\frac{1}{{d_{y} }}} & {v_{0} } \\ 0 & 0 & 0 \\ \end{array} } \right]\left[ {\begin{array}{*{20}c} f & 0 & 0 & 0 \\ 0 & f & 0 & 0 \\ 0 & 0 & 1 & 0 \\ \end{array} } \right]\left[ {\begin{array}{*{20}c} R & t \\ {0^{\text{T}} } & 1 \\ \end{array} } \right]\left[ \begin{aligned} X \\ Y \\ Z \\ 1 \\ \end{aligned} \right].$$

The matrix $$K = \left[ {\begin{array}{*{20}{l}}{\frac{f}{{{d_x}}}}&0&{{u_0}}&0\\0&{\frac{f}{{{d_y}}}}&{{v_0}}&0\\0&0&1&0\end{array}} \right]$$ was referred to as the camera internal reference matrix, which was only related to the camera. $$T_{\text{CW}} = \left[ {\begin{array}{*{20}c} R & t \\ {0^{\text{T}}} & 1 \\ \end{array}} \right]$$ was the external parameter matrix of the camera. $P = KT_{\text{CW}}$ was the perspective projection matrix.

Two kinds of distortions have a considerable influence on the projected image: radial distortion and tangential distortion, respectively [64]. In this paper, the Taylor series was used to correct the radial distortion, and rectangular projection imaging was used to correct the tangential distortion.

1. Radial distortion
  In general, the radial distortion at the centre of the imager was 0. As it moved towards the edge, the radial distortion became increasingly serious. However, the radial distortion could be corrected by the following Taylor series expansion:
  $$X_{\text{C}} = x(1 + K_{1} r^{2} + K_{2} r^{4} + K_{3} r^{6} )$$
  $$Y_{\text{C}} = y(1 + K_{1} r^{2} + K_{2} r^{4} + K_{3} r^{6} )$$
  (*x*, *y*) was the original position of the distortion point on the imager. *r* was the distance from the point to the centre of the imager. $\left( {X_{\text{C}} ,Y_{\text{C}} } \right)$ was the new position after correction.
2. Tangential distortion
  When the imager was attached to the camera, a certain error was produced. The plane of the image was not completely parallel to the lens, which caused tangential distortion. Tangential distortion could be corrected by the following formula:
  $$X_{\text{C}} = x + \left[ {2P_{1} y + P_{2} (r^{2} + 2x^{2} )} \right]$$
  $$Y_{\text{C}} = y + \left[ {2P_{2} x + P_{1} (r^{2} + 2y^{2} )} \right].$$
  (*x*, *y*) was the original position of the distortion point on the imager. *r* was the distance from the point to the centre of the imager. $\left( {X_{\text{C}} ,Y_{\text{C}} } \right)$ was the new position after correction.

#### Three-dimensional registration

Three-dimensional registration was the core in AR and the most important step to implement the AR navigation system. The key issue was estimating the pose of the vision sensor in a 3D environment and finding objects in the scene [65]. A rigid connection was established between the virtual model and the logo. In this way, the virtual model could be moved to the desired location by moving the logo. In this process, the positional relationship between the logo and the virtual model was considered constant. Extracting 3D models from preoperative images was necessary to enhance the endoscopic view of the surgical scene [66]. Therefore, in the process of realizing AR, obtaining CT data of the model and performing 3D reconstruction of the model based on CT data was necessary. The reconstructed 3D model was then imported into the video stream. The virtual model was matched with the real model registration in the camera video stream by moving the logo.

**Fig. 8:**
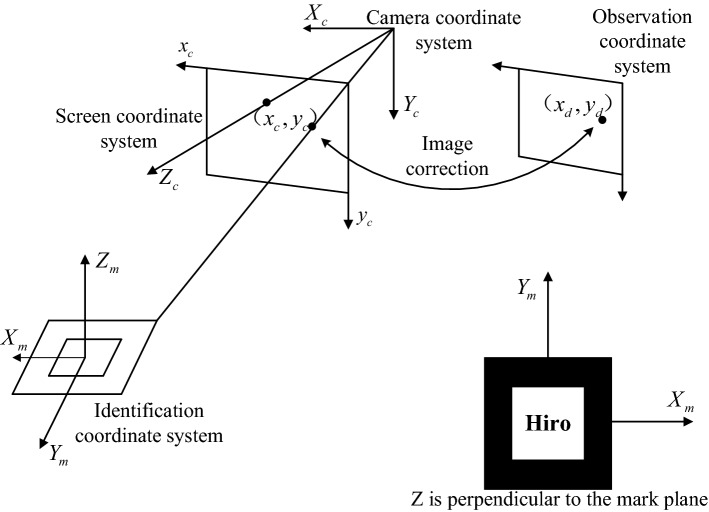
Conversion between coordinate systems in the ARToolKit

The correlation between the coordinate systems was the key to realizing the 3D registration of a virtual object and real scene object. The coordinate system used by ARToolKit is shown in Fig. 8. The observation coordinate system was a 2D coordinate system [67].

**Fig. 9:**
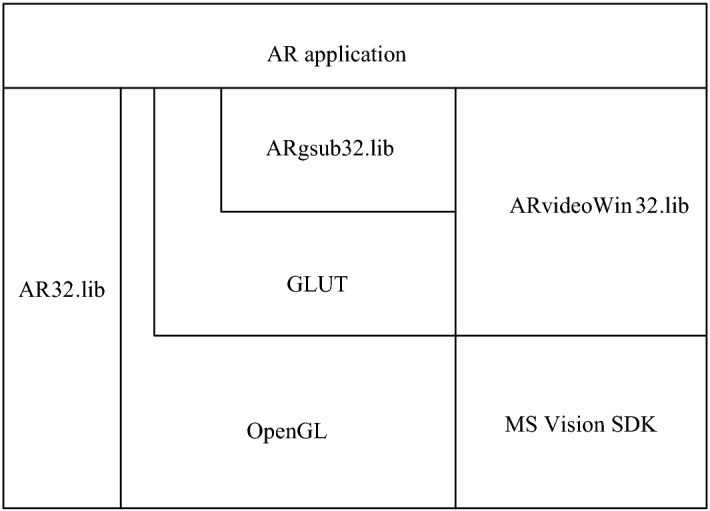
ARToolKit architecture

The entire experimental software system was implemented using the Microsoft Foundation class in conjunction with ARToolKit programming. ARToolKit was open source, with a simple configuration and simple documentation and was primarily used in many AR applications. The programming used computer vision technology to calculate the position and pose of the observer’s viewpoint relative to a known marker and supported AR applications that are based on vision or video. ARgsib.lib was based on the graphics processing function library. ARToolKit primarily consisted of several function libraries as shown in Fig. 9.

### AR registration experiment

#### Virtual and real registration

**Fig. 10:**
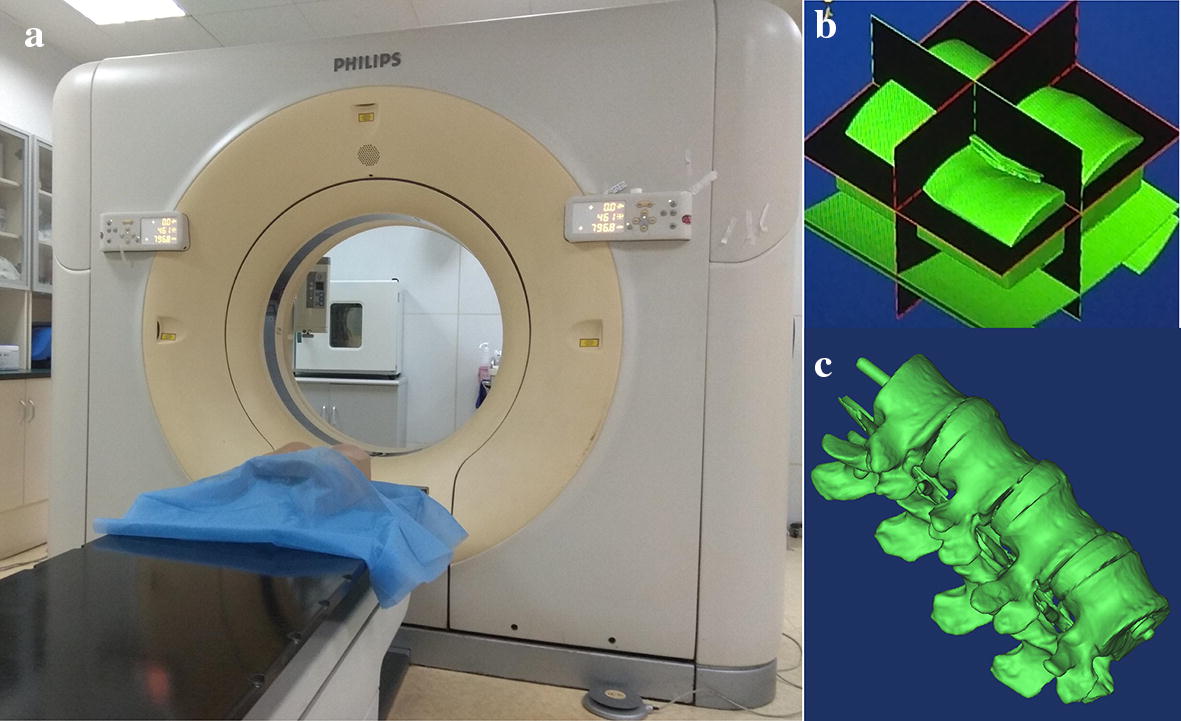
**a** Spinal model was scanned by CT; **b** 3D reconstruction of spinal puncture model; **c** 3D reconstruction model of the spine after removing the skin and flesh

**Fig. 11:**
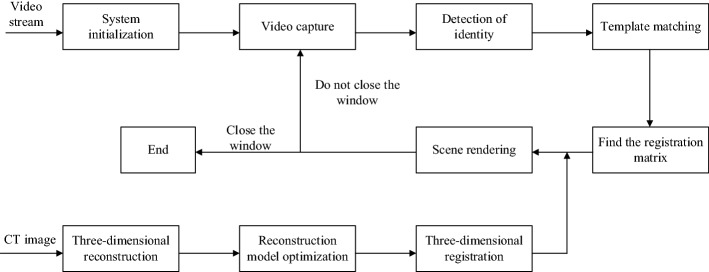
Process of registration experiment

The virtual and real registration primarily achieved the registration of the preoperative patient’s imaging data with the actual surgical scene during the operation [68]. In the registration process, the relative positional relationship between the camera and the logo was calculated in real time. As shown in Fig. 10, CT images of the spinal model were obtained, and 3D reconstruction was performed on the CT images to obtain the virtual model. The 3D reconstruction of the spinal model was processed, and necessary materials and lighting were added to make the display more realistic. The model needed to be more transparent to ensure that it could display internal lesion information. The flesh of the model surface was removed by a transparent method, which provided a clearer view of the internal spinal model. The camera was connected to the workstation, and the video stream collected by the camera was displayed to the user in real time in the system. The identification method of the ARToolKit Software Development Kit (SDK) was used to identify the prepared logo [69]. The 3D virtual model was displayed in the location where the logo is fixed. In this way, the 3D model was moved to match the real model by moving the logo. The specific AR registration experimental flow chart is shown in Fig. 11.

**Fig. 12:**
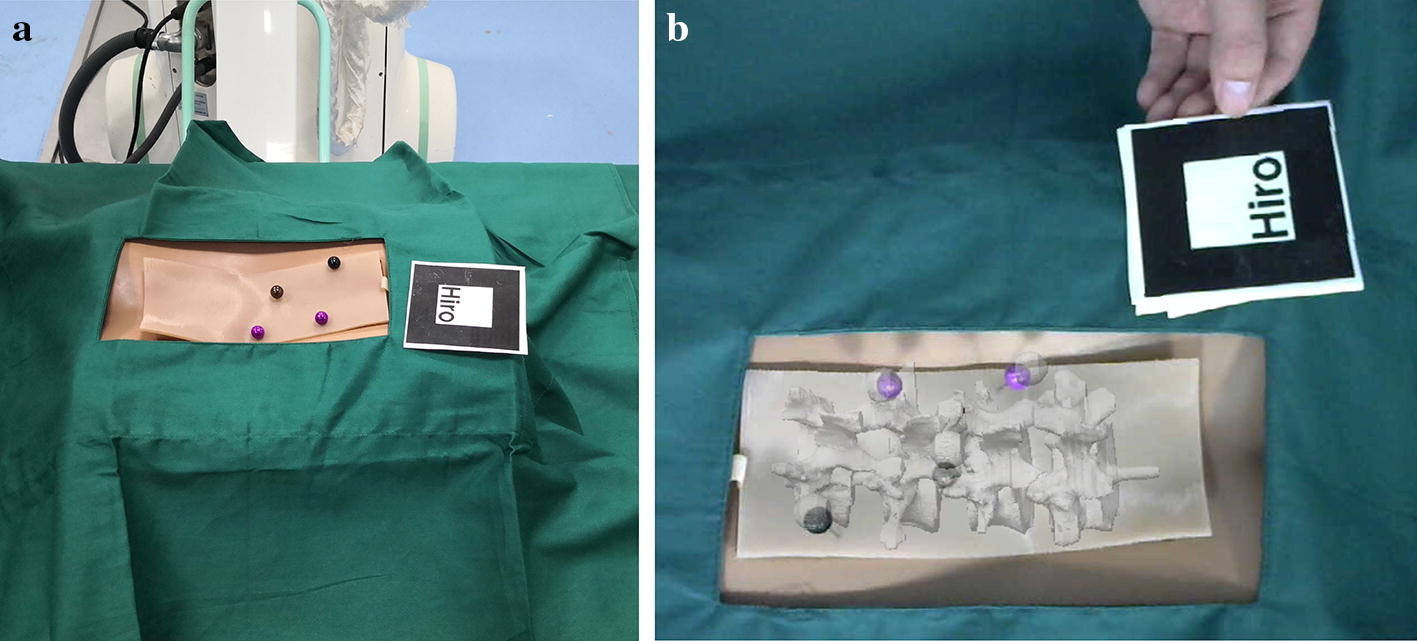
Virtual and real registration is performed by moving a logo. **a** An original spinal puncture model and a logo; **b** virtual model and real model are superposed by moving a logo

In the experiment, the model and logo were placed on the workbench and kept within the camera’s field of view. The program was then run to import the optimized virtual spinal model into the video stream captured by the camera. The virtual model and the real model were overlapped by moving a logo. Subsequently, the posture and position of the spinal model were constantly changed to perform experiments of virtual and real registration. As shown in Fig. 12, the experiment was repeated and the experimental results were verified.

#### Error calculation

**Fig. 13:**
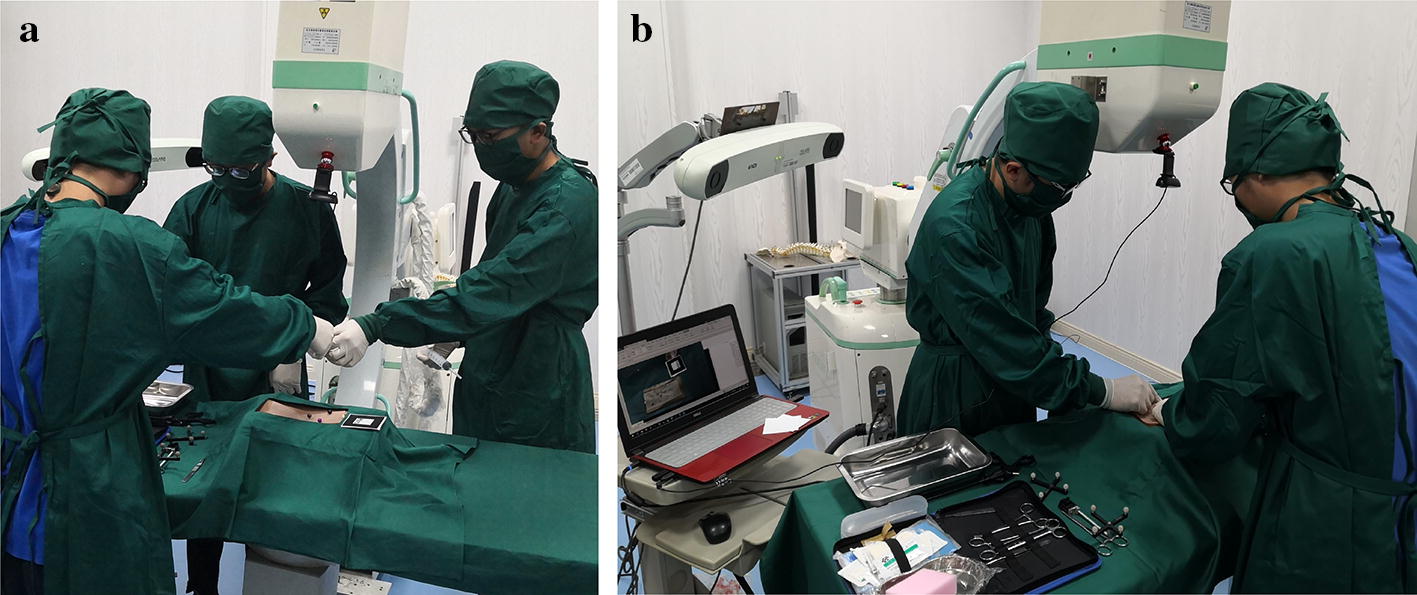
**a** Preparation before the experiment; **b** position of the small ball is taken by the probe point, and the virtual coordinate value of the small ball l is acquired by using the NDI

The NDI light spheres were employed as markers to attach to the model of the spine. After the virtual model was registered with the real model, the coordinate values of each marker point were read and recorded in real time. As shown in Fig. 13a, we needed to prepare for the experiment. At this point, the logo had to be kept stationary to cover the real model to ensure that the real model did not appear in the video stream. Only the virtual model remained and the tip of the probe was pointed to the mark of the virtual model.

**Fig. 14:**
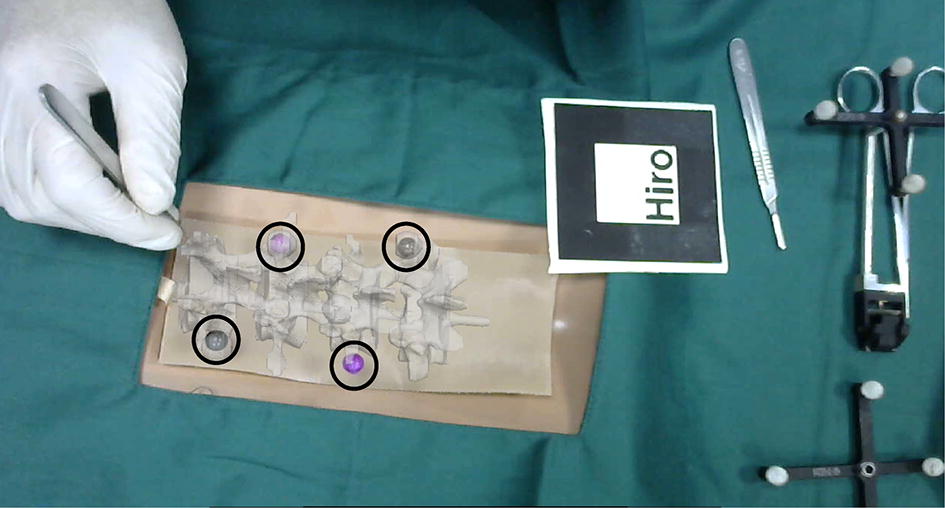
Virtual and real registration image of the spine model

In the process of registration, mark points could be used as reference points for registration of the model. After the registration was completed, the coordinate value of the NDI ball on the probe could be read in real time via the NDI optical tracking system. In this way, the coordinate of the tip of the probe was calculated and the value of this coordinate was recorded. The real model then reappeared in the video stream as shown in Fig. 13b. At this point, the NDI optical tracking system could be used to read the positions of the balls that were attached to the model and calculate their coordinate values in the world coordinate system. The error of the registration was calculated by comparing the values of the previously recorded coordinates. The first step was to read the position of the NDI sphere that was fixed to the model in NDI coordinates. The second step was to run the program and move the logo to ensure that the points on the virtual model coincide with those on the real model. As shown in Fig. 14, the virtual model was superimposed on the real model, and the virtual and real registration was performed by four small balls. The virtual spinal model was superimposed on the real spinal model. At this point, the final registration of the virtual model and real model was completed. The logo was kept stationary while moving the real model away. Last, the real model could not appear in the video stream and leaving only the model of the virtual spine remained. Each corresponding mark point on the virtual model was taken with a probe point. The position of the probe read by the NDI system was recorded. According to the position of the probe, the coordinate of the position of the tip of the probe could be calculated.

The posture of the spinal model was changed and the coordinates of the NDI ball were reread. The experiment was continued and the data were recorded according to the experimental procedure. Four sets of independent experiments were carried out to repeat the experimental procedures, and four sets of data were obtained. The errors of registration were separately calculated. The coordinates of four marking points on the model in the NDI coordinate system were listed. Specific experimental data are shown in Table 1.

#### Calculation of registration error after improved identification method

**Fig. 15:**
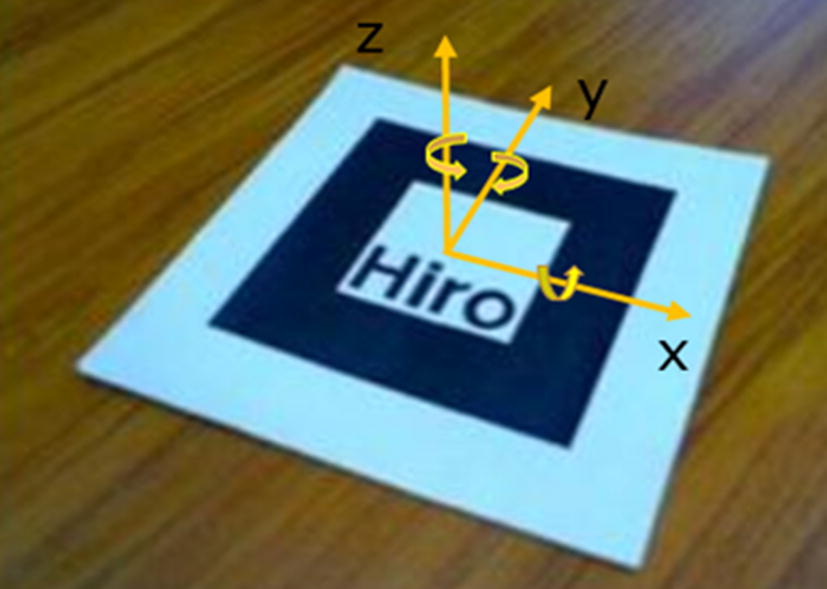
Software control model movement and rotation

As shown in Table 1, the error of the virtual and real registration was relatively large; thus, the experimental method had to be optimized. The method based on improved identification was implemented to reduce the experimental error as much as possible. Achieving accurate results was very difficult by the operation of adjusting the logo. Software was implemented to control the movement and rotation of the virtual model. In the registration process, the virtual model was moved to the position of the real model in space based on the general identification method. After the first registration, the 3D model was rotated with six degrees of freedom to achieve the second accurate registration. As shown in Fig. 15, the single movement and rotation was 1 mm and $1^{^\circ }$, respectively, to ensure accuracy.

**Fig. 16:**
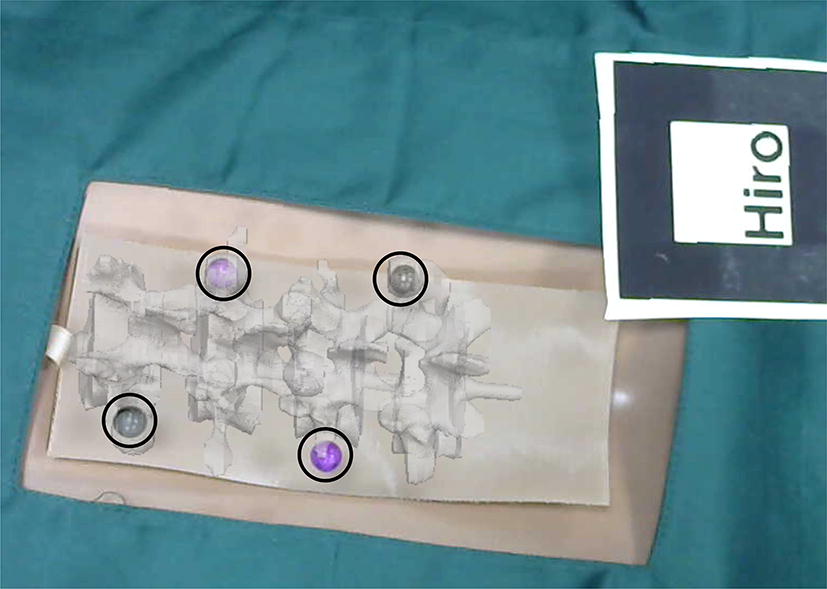
Movement and rotation of the virtual model based on software control

At this time, we would perform the registration experiment again. The improved program had to be run to register the virtual model with the real model in the same way. In many cases, adjusting the logo to make the virtual model and the real model completely coincide with the best results was more difficult. Repeated experiments revealed that the virtual model and the real model could hardly be completely overlapped by the general identification method when the model was in some positions. After the registration based on the general identification method was completed, the logo was kept stationary. At this time, the keyboard was needed to input instructions to make the virtual model move or rotate, and then the second registration was carried out until the ideal effect was achieved. The experimental results are shown in Fig. 16.

According to the above experimental procedure, the virtual and real registration was carried out by using general identification method combined with software control. Four different positions and attitudes of the models were tested independently, and four sets of data were obtained. The experimental data are shown in Table 2.

#### Calculation of registration error based on robot-assisted method

The error of the virtual and real registration was substantially reduced by using the improved identification method. The improvement of effect was distinct and the accuracy of virtual and actual registration was considerably improved. However, the current error was not satisfactory, and the requirements of high precision were not satisfied in the actual operation process.

**Fig. 17:**
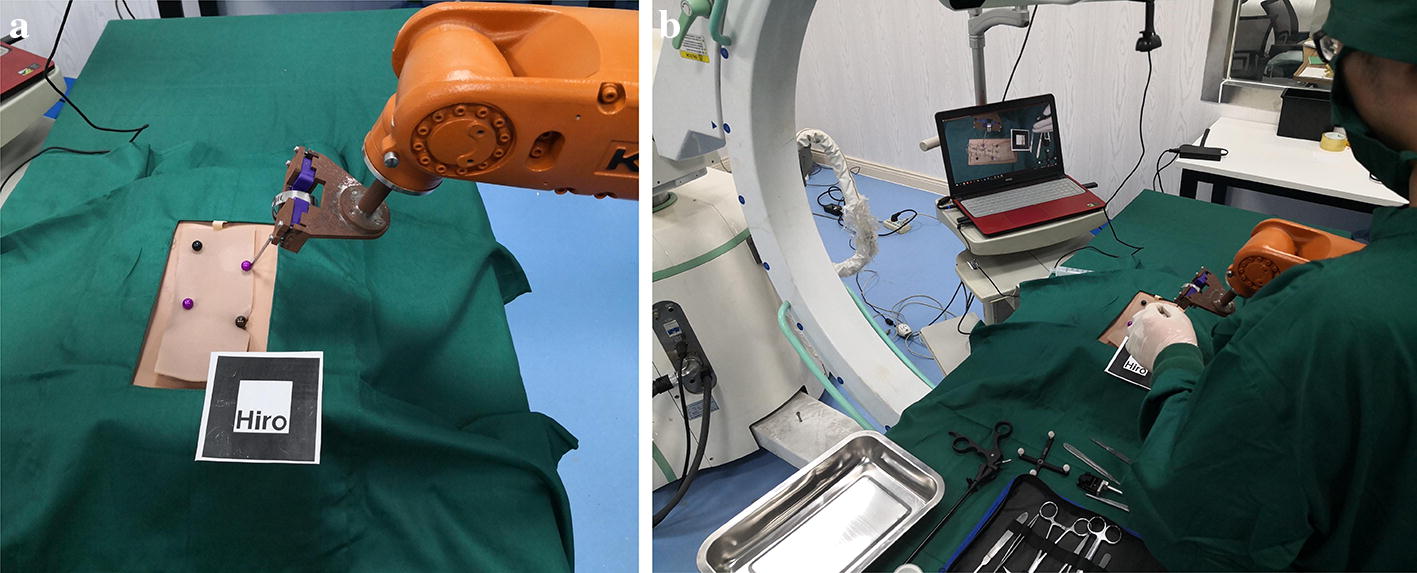
**a** Image of robot puncture effect; **b** observation of puncture effect

During the experiment, the method of picking up markers on the virtual model by probe was been adopted by previous experiments. When a person picked up a probe to take a mark on a virtual model, a large error was produced due to problems such as hand tremor and lack of depth information of the human eye. Therefore, the method of using a robot to puncture the spinal surface instead of human hands was adopted by this experiment. The robot could be used to pick the mark points on the virtual model. The specific measures are described as follows: first, the virtual and real registration of the spine model was performed by the previous improved identification method. Second, after the virtual and real registration was completed, the operator started to operate the KUKA robot instructor to manipulate the KUKA robot for puncture. When the KUKA robot moved to the mark point, the robot performed puncture. Last, the previous method was adopted and the real model was removed. The robot was used to take the marker point of the virtual model, and the position data of probe tip read by the NDI optical tracking system was recorded. According to the position number of the probe, the tip coordinate could be calculated. The experimental effects are shown in Fig. 17a. As shown in Fig. 17b, the experimental procedure was repeated to perform four independent experiments, and four sets of data were obtained. Their registration errors were separately calculated. The experimental results are shown in Table 3. The robot was used instead of the human hand for puncture. After the marker point was taken, the average error of the virtual and real registration ranged between 2.39 and 2.58 mm, which proved that our improved measures achieved satisfactory results.

## Data Availability

The authors agree to make all published data available.

## Abbreviations

CAS: computer-aided surgery
IGS: image-guided surgery
2D: two-dimensional
3D: three-dimensional
AR: augmented reality
VR: virtual reality
NDI: Northern Digital Inc
VS 2010: Visual Studio 2010
MFC: Microsoft Foundation Classes
SDK: Software Development Kit
